# Differences in Rotavirus Shedding and Duration by Infant Oral Rotavirus Vaccination Status in Dhaka, Bangladesh, 2011–2014

**DOI:** 10.1093/infdis/jiad502

**Published:** 2023-11-29

**Authors:** Jenna Ciszewski, Mami Taniuchi, Benjamin Lee, E Ross Colgate, James A Platts-Mills, Rashidul Haque, K Zaman, Benjamin Lopman, William A Petri, Beth D Kirkpatrick, Elizabeth T Rogawski McQuade

**Affiliations:** Department of Epidemiology, Emory University, Atlanta, Georgia, USA; Division of Infectious Diseases and International Health, University of Virginia, Charlottesville, Virginia, USA; Department of Biomedical Engineering, University of Virginia, Charlottesville, Virginia, USA; Department of Civil and Environmental Engineering, University of Virginia, Charlottesville, Virginia, USA; Department of Pediatrics, Larner College of Medicine, University of Vermont, Burlington, Vermont, USA; Department of Microbiology and Molecular Genetics, Vaccine Testing Center, Larner College of Medicine, University of Vermont, Burlington, Vermont, USA; Division of Infectious Diseases and International Health, University of Virginia, Charlottesville, Virginia, USA; International Centre for Diarrheal Disease Research, Dhaka, Bangladesh; International Centre for Diarrheal Disease Research, Dhaka, Bangladesh; Department of Epidemiology, Emory University, Atlanta, Georgia, USA; Division of Infectious Diseases and International Health, University of Virginia, Charlottesville, Virginia, USA; Department of Microbiology and Molecular Genetics, Vaccine Testing Center, Larner College of Medicine, University of Vermont, Burlington, Vermont, USA; Department of Epidemiology, Emory University, Atlanta, Georgia, USA

**Keywords:** rotavirus, diarrhea, enteric infections, vaccination

## Abstract

To evaluate how breakthrough rotavirus disease contributes to transmission, we examined the impact of rotavirus vaccination on fecal shedding and duration of illness. We used multivariable linear regression to analyze rotavirus quantity by RT-qPCR and duration among 184 episodes of rotavirus diarrhea positive by ELISA in the PROVIDE study. Vaccinated children had less fecal viral shedding compared to unvaccinated children (mean difference = −0.59 log copies per gram of stool; 95% confidence interval [CI], −.99 to −.19). Duration of illness was on average 0.47 days (95% CI, −.23 to 1.17 days) shorter among vaccinated children. Rotarix vaccination reduces shedding burden among breakthrough cases of rotavirus gastroenteritis.

***Clinical Trials Registration***. NCT01375647.

## Teaser

We estimated the effect of rotavirus vaccination on duration and quantity of rotavirus shed during rotavirus gastroenteritis in Bangladesh. Virus quantity was lower in symptomatic vaccinated children compared to symptomatic unvaccinated children, but differences in episode duration were small.

Rotavirus is a leading cause of diarrhea in children, accounting for an estimated 128 500 deaths in 2016 [1]. The introduction of oral rotavirus vaccines, recommended by the World Health Organization in 2009 [2], has contributed to marked reductions in rotavirus morbidity and mortality [3]. However, rotavirus vaccines are less effective in low-income settings, where child mortality and rotavirus disease burden are greatest [4, 5]. A meta-analysis of rotavirus vaccine effectiveness (VE) found that Rotarix VE among children <1 year of age was 63% in settings with high child mortality compared to 86% in settings with low child mortality [5]. Because few studies have examined the impact of rotavirus vaccination on fecal shedding and infectiousness, it is unknown how much breakthrough cases (ie, cases occurring postvaccination), which are common in low-resource settings, contribute to transmission.

Vaccination may impact quantity of rotavirus shed and, by extension, infectiousness (ie, likelihood of transmission to contacts) by preventing cases of rotavirus gastroenteritis (RVGE) from occurring or by converting symptomatic episodes to lower-burden asymptomatic infections [6]. Because rotavirus shedding generally peaks in the first few days of symptom onset but persists past symptom resolution [7–9], vaccination could also reduce the quantity of virus shed in the environment by suppressing viral load and/or shortening the duration of shedding among breakthrough cases. While previous studies from India and Malawi have characterized general rotavirus shedding patterns, these studies had little to no variability in vaccination status among participants [7, 8, 10]. The impact of rotavirus vaccination on fecal shedding among breakthrough cases is largely unknown.

In the Performance of Rotavirus and Oral Polio Vaccines in Developing Countries (PROVIDE) randomized controlled clinical trial conducted in the Mirpur slum of Dhaka, Bangladesh [11], per protocol vaccine efficacy was 51% against all rotavirus diarrhea and 73.5% against severe rotavirus diarrhea, resulting in a substantial number of breakthrough RVGE episodes [12]. Among infants in PROVIDE, we examined the relationship between vaccination status and (1) quantity of rotavirus shed during episodes of RVGE, and (2) duration of illness to enhance our understanding of the impacts of vaccination on transmission.

## METHODS

We used data from the PROVIDE randomized controlled clinical trial conducted from May 2011 to November 2014, previously described [11, 12]. Seven hundred infants were enrolled in the first week of life and followed until age 2 years. The children were randomized to receive 2 doses of Rotarix vaccine at 10 and 17 weeks of age or no vaccine. Informed consent was obtained for all participants. Human subjects research approval was obtained from the Ethics Review Committee and the Research Review Committee at the International Centre for Diarrhoeal Disease Research, Bangladesh (icddr, b) and from the institutional review boards at the University of Virginia and University of Vermont. PROVIDE was registered at Clinicaltrials.gov (NCT01375647).

Field research assistants conducted twice weekly home visits to identify diarrhea defined as 3 or more abnormally loose stools in 24 hours according to the mother [11] separated by 72 diarrhea-free hours. One fecal sample was collected as soon as possible [12]. Stool samples were tested for the presence of rotavirus by ProsSpecT enzyme-linked immunosorbent assay (ELISA; Oxoid). Independent of the ELISA results, nucleic acid was extracted from all fecal samples collected in the first year of life and analyzed by quantitative reverse transcription polymerase change reaction (RT-qPCR) using the TaqMan Array Card platform (ThermoFisher). Rotavirus was detected using the nonstructural protein *NSP3* gene [13]. Episodes with missing stool samples were presumed negative for rotavirus [12] and excluded.

Consistent with the original trial [11], an episode of RVGE was defined as diarrhea positive for rotavirus by ELISA. In sensitivity analysis, RVGE was defined if the stool sample tested rotavirus positive by RT-qPCR at any cycle threshold (Ct) value of <35, regardless of ELISA result. The primary outcomes of interest were quantity of rotavirus (viral load) shed in stool and duration of illness (days) during episodes of RVGE. The exposure was determined by Rotarix randomization arm and analyzed per intention-to-treat. Fecal viral load was measured in log-copy numbers per gram of stool (log base 10) based on the raw Ct values from RT-qPCR using the equation (35 − Ct)/3.322. Only episodes of RVGE in children between the ages of 10 weeks and 1 year were included, because children randomized to receive Rotarix were not administered their first dose until 10 weeks and episodes after 1 year were not tested by RT-qPCR. All children were included regardless of natural rotavirus infection before 10 weeks.

Because we restricted the study population on postrandomization factors (ie, occurrence of RVGE), we adjusted for potential confounding variables that were selected a priori based on a causal framework constructed following a literature review. Variables included were disease severity, child age, nutritional status, number of days of exclusive breastfeeding, and time (days) from symptom onset to stool collection. Time from symptom onset to stool collection was not included in the model for duration of illness only, due to its correlation with duration. RVGE severity was classified using the 20-point modified Vesikari scale, with a score of ≥ 11 indicating severe disease [14]. Child age in days was continuous when used as an adjustment variable in the model, but was categorized to produce stratified estimates (10 weeks to 6 months vs 6 to 12 months). Nutritional status was evaluated using anthropometric measures to calculate weight-for-age (WAZ) and height-for-age (HAZ) z-scores [11] modeled as continuous variables. We classified children as stunted (HAZ) or underweight (WAZ) if their z-score was less than −2.

All statistical analyses were performed using SAS 9.4. χ^2^ and ANOVA tests were conducted to compare participant characteristics across groups. *P* values < .05 were considered statistically significant. Mean differences in Table 1 and Table 2 were calculated using multivariable linear regression (MLR). MLR was also used to investigate the association between vaccination and (1) fecal viral shedding quantity and (2) duration of illness during episodes of RVGE. Generalized estimating equations with robust standard errors were used to account for clustering of RVGE episodes in the same child and nonnormality of the outcomes.

**Table 1.** Quantity of Fecal Shedding (by RT-qPCR) by Episode and Child Characteristics Among Cases of RVGE (Positive by ELISA)

	Shedding Quantity, Log_10_ Copies per Gram of Stool				
Characteristic	No. of Episodes	Median	IQR	Range	Mean Difference (95% CI)^a^
Randomization group					
Vaccine	65	7.71	6.85–8.74	3.71–10.75	…
Placebo	119	8.42	7.67–9.08	4.60–11.00	…
Episode attributes					
Severity (Vesikari score)^b^					
Mild (< 7)	44	8.53	7.68–9.20	5.25–10.53	Ref
Moderate (7–10)	57	7.94	7.24–8.77	3.71–10.11	−0.42 (−.90 to .06)
Severe (≥11)	48	8.24	7.42–9.45	4.35–11.00	−0.25 (−.81 to .31)
Time from symptom onset					
0–1 d	93	8.23	7.59–9.08	4.35–10.75	Ref
2–3 d	67	8.13	7.51–8.96	3.71–11.00	−0.08 (−.55 to .39)
≥ 4 d	24	8.47	7.19–8.86	5.34–10.53	−0.16 (−.84 to .51)
Child attributes					
Child age at episode					
10 wk to 6 mo	63	7.99	7.01–8.76	4.60–10.53	−0.48 (−.96 to −.00)
6 mo to 1 y	121	8.27	7.64–9.08	3.71–11.00	Ref
Underweight (WAZ < −2)					
Yes	32	8.44	7.37–9.28	4.35–10.53	0.33 (−.32 to .98)
No	152	8.20	7.56–8.89	3.71–11.00	Ref
Stunted (HAZ < −2)					
Yes	26	8.37	7.47–9.95	4.35–10.53	0.12 (−.66 to .91)
No	158	8.20	7.55–8.90	3.71–11.00	Ref
Exclusive breastfeeding at episode					
Yes	14	8.72	7.55–9.30	5.59–10.04	0.54 (−.38 to 1.46)
No	170	8.20	7.55–8.92	3.71–11.00	Ref

Abbreviations: CI, confidence interval; ELISA, enzyme-linked immunosorbent assay; HAZ, height-for-age z-score; IQR, interquartile range; RT-qPCR, quantitative reverse transcription polymerase change reaction; RVGE, rotavirus gastroenteritis; WAZ, weight-for-age z-score.

^a^Adjusted for the other covariates in the table.

^b^Missing for 35 episodes due to missing dehydration status.

**Table 2.** Association of Vaccination With Quantity of Fecal Viral Shedding by RT-qPCR and With Duration of Illness in Days

Analysis	No.	Quantity of Fecal Shedding^a^		Duration of Illness^b^	
		Mean Difference (95% CI), Log Copies per Gram of Stool	*P* Value for Heterogeneity	Mean Difference, d (95% CI)	*P* Value for Heterogeneity
Effect of vaccination	184	−0.59 (−.99 to −.19)	…	−0.47 (−1.17 to .23)	…
Effect modification by severity					
Mild	44	−0.48 (−1.19 to .24)	…	−0.44 (−1.19 to .32)	…
Moderate	57	−0.85 (−1.52 to −.18)	.46	−0.32 (−1.47 to .83)	.86
Severe	48	−0.66 (−1.67 to .35)	.77	1.04 (−.69 to 2.76)	.13
Effect modification by age					
< 6 mo	63	−0.73 (−1.40 to −.07)	…	−0.87 (−2.25 to .52)	…
≥ 6 mo	121	−0.50 (−1.01 to .003)	.58	−0.27 (−1.10 to .55)	.48
Effect modification by doses received					
10 wk–19 wk	22	−1.40 (−2.48 to −.31)	…	−0.35 (−2.85 to 2.15)	…
19 wk–1 y	162	−0.51 (−.96 to −.06)	.13	−0.51 (−1.25 to .23)	.90

Abbreviations: CI, confidence interval; HAZ, height-for-age z-score; RT-qPCR, quantitative reverse transcription polymerase change reaction; WAZ, weight-for-age z-score.

^a^Controlling for age, WAZ, HAZ, exclusive breastfeeding at time of episode, and time (days) since symptom onset.

^b^Controlling for age, WAZ, HAZ, and exclusive breastfeeding at time of episode.

In addition, we used interaction terms to investigate potential effect modification by disease severity (modified Vesikari score), child age (10 weeks to 6 months vs 6 to 12 months), and number of vaccine doses received (1 dose at week 10 vs 2 doses at week 19). We chose 19 weeks as the cutoff to allow a 2-week period for children to mount an immune response to the second dose.

## RESULTS

In total, 700 children were enrolled in PROVIDE, and 350 were randomized to each vaccine arm. Of these, 107 (15.3%) were lost from the study within the first year, 58 in the vaccine arm and 49 in the control arm [12]. Nine episodes of natural rotavirus infection occurred in children <10 weeks of age. Of these, 7 (78%) were among children randomized to the vaccine arm (*P* = .09). Of vaccinated children, 17.8% (n = 62) experienced at least 1 episode of RVGE between the ages of 10 weeks and 1 year, compared with 32.0% (n = 112) of unvaccinated children (Supplementary Table 1). We found no significant differences in child characteristics across groups (Supplementary Table 1).

In 184 episodes of RVGE detected by ELISA, quantity of fecal viral shedding ranged from 3.71 to 11.00 log copies per gram of stool, measured by RT-qPCR. Of these, 65 episodes (35%) occurred among vaccinated children and 119 (65%) occurred among control children. Median viral load was 7.71 (interquartile range [IQR], 6.85–8.74) log copies per gram of stool for episodes of RVGE occurring in vaccinated children and 8.42 (IQR, 7.67–9.08) log copies per gram of stool for episodes occurring in control children (Table 1).

The average fecal viral shedding during episodes of RVGE was 0.59 (95% confidence interval [CI], .19–.99) log copies per gram of stool lower among vaccinated as compared to unvaccinated children, after controlling for age, WAZ, HAZ, exclusive breastfeeding at time of episode, and time in days since symptom onset (Table 2). Children in the vaccine arm also had a shorter duration of illness by 0.47 days (95% CI, −0.23 to 1.17) days than children in the control arm (Table 2). However, the duration results were not statistically significant. We found no evidence of statistically significant heterogeneity for the effect of vaccination on quantity of fecal shedding or duration of illness (Table 2) by disease severity, age, or time interval (10 to 19 weeks vs 19 weeks to 1 year).

In the sensitivity analysis, where we included all diarrhea episodes positive for rotavirus by RT-qPCR (n = 377), fecal viral shedding during episodes of RVGE was 0.79 (95% CI, .41–1.17) log copies per gram of stool lower among vaccinated as compared to unvaccinated children (Supplementary Table 2). The sensitivity analysis for the duration outcome showed variable and imprecise results, with no overall effect of vaccination on duration among all RT-qPCR positive episodes (mean difference, −0.09 days; 95% CI, −.71 to .54; Supplementary Table 3).

## DISCUSSION

In a birth cohort of Bangladeshi children 10 weeks to 1 year of age, lower quantities of virus were detected in stools from vaccinated children with symptomatic rotavirus infection than in stools from symptomatic unvaccinated children. This relationship did not differ by child age or disease severity. In addition, symptomatic vaccinated children had modest reductions (approximately half a day) in disease duration compared to symptomatic unvaccinated children, but these results were not statistically significant or consistent in sensitivity analysis.

These results suggest that Rotarix vaccination reduces shedding quantity among breakthrough cases of RVGE in addition to preventing RVGE cases entirely. Assuming quantity of rotavirus detected by RT-qPCR is a reasonable proxy for the quantity of infectious virus shed, breakthrough cases among vaccinated children may have lower transmission potential than cases among unvaccinated children. However, studies that directly estimate vaccine efficacy against transmission are needed to determine if lower shedding among vaccinated children leads to lower infectiousness. For example, a study of Malawian children found that transmission to household contacts was lower among less severe vaccinated cases compared to severe vaccinated cases, suggesting that vaccine-induced reductions in severity of disease translates to reduced transmission [15].

Because previous studies have reported higher shedding among children with severe disease compared to children with mild or moderate disease, we stratified our analysis on disease severity [7, 10]. We found no evidence of effect modification by severity; however, it should be noted that the vaccine prevented most cases of severe diarrhea in the vaccinated children from occurring [12].

Our analysis is subject to several limitations. First, because stool specimens in the full cohort were only tested by RT-qPCR for diarrhea episodes, we were unable to examine shedding among asymptomatic infections. Quantity of virus shed and/or transmission from asymptomatic children may or may not differ from that in symptomatic children and could not be evaluated here. Second, ELISA-positive episodes occurring near the time of vaccine administration could have resulted from shedding of the vaccine. However, the effect was consistent when restricting the analysis to episodes between 19 weeks to 1 year of life, and we found no clustering of episodes around vaccination events (Supplementary Figure 1). Third, because ELISA has limited sensitivity, true rotavirus infections with low-level viral shedding were likely missed, with breakthrough cases likely being more subject to this misclassification. However, the sensitivity analysis among all rotavirus RT-qPCR–positive episodes found similar results. Fourth, only 1 stool sample was collected per episode at varying times from symptom onset. Serial stool samples at predetermined intervals would have allowed for analysis of shedding patterns throughout the illness course. Finally, although RT-qPCR is highly sensitive, it is unable to distinguish between live and dead virus particles, and our ability to make inferences about transmission is limited. Similarly, the effects on illness duration may not translate to effects on duration of transmissibility because shedding generally peaks in the first few days of symptoms but persists past symptom resolution [7–9].

## CONCLUSIONS

In addition to preventing disease, rotavirus vaccination reduces the quantity of virus shed in stool during episodes of RVGE. While viral load is an important determinant of secondary rotavirus transmission, additional studies are needed to determine its impact on community-level transmission dynamics.

## Supplementary Data

Supplementary materials are available at *The Journal of Infectious Diseases* online (http://jid.oxfordjournals.org/). Supplementary materials consist of data provided by the author that are published to benefit the reader. The posted materials are not copyedited. The contents of all supplementary data are the sole responsibility of the authors. Questions or messages regarding errors should be addressed to the author.

## Supplementary Material

jiad502_Supplementary_Data

## Notes

***Author contributions*.** J. C. conducted the analysis and drafted the manuscript. J. C., B. Lopman, and E. T. R. M. conceived of the project. M. T. led the laboratory analyses and J. A. P.-M. analyzed laboratory data. B. Lee, E. R. C., and B. D. K. led the parent study. All authors contributed to the analytic plan and critically reviewed the manuscript.

***Financial support*.** This work was supported by the National Institute of Allergy and Infectious Disease at the National Institutes of Health (grant number K01AI130326 to E. T. R. M.). PROVIDE was funded by the Bill and Melinda Gates Foundation (grant number OPP1017093 to W. A. P.).
