# Supplementary material for: Differences in Rotavirus Shedding and Duration by Infant Oral Rotavirus Vaccination Status in Dhaka, Bangladesh, 2011–2014
Source: J Infect Dis. 2023 Nov 29;230(1):e75–9. doi: 10.1093/infdis/jiad502 (PMC11272065; doi:10.1093/infdis/jiad502)

Supplemental Materials

Differences in Rotavirus Shedding and Duration by Infant Oral Rotavirus Vaccination Status in Dhaka, Bangladesh 2011 - 2014

Table S1. Child characteristics by vaccination arm and rotavirus diarrhea defined by detection by ELISA in the first year of life in PROVIDE

Characteristic	Rotarix, breakthrough RV diarrhea (n=62)		Rotarix, no breakthrough RV diarrhea (n=288)		No Rotarix, RV diarrhea (n=112)		No Rotarix, no RV diarrhea (n=238)	
	mdn	*IQR*	*mdn*	*IQR*	*mdn*	*IQR*	*mdn*	*IQR*
**Child attributes**								
Sex, Male (n,%)	40	64.5%	142	49.3%	61	54.5%	125	52.5%
Median age at enrollment (days)	5	4, 6	5	3, 6	5	3.5, 6	6	4, 7
Weight at enrollment (kg)	2.8	2.5, 2.9	2.7	2.5, 3.0	2.7	2.5, 3.0	2.8	2.5, 3.1
Length at enrollment (cm)	48.5	47.2, 49.9	48.5	47.3, 49.6	48.7	47.5, 50.0	48.9	47.5, 50.1
Has $\geq$ 1 siblings <5 years (n,%)	19	30.6%	77	26.7%	33	29.5%	59	24.8%
Weight-for-age z score at 10 wk[a]	-1.09	-1.82, -0.54	-0.87	-1.60, -0.34	-0.96	-1.54, -0.42	-0.84	-1.50, -0.16
Height-for age z score at 10 wk[a]	-1.11	-1.53, -0.48	-0.97	-1.63, -0.45	-0.98	-1.49, -0.44	-0.84	-1.62, -0.34
Weight-for-height z score at 10 wk[a]	-0.14	-1.22, 0.55	-0.08	-0.62, 0.63	-0.03	-0.81, 0.62	0.07	-0.65, 0.69
Exclusively breastfed at 18 wk[b] (n,%)	28	45.9%	126	51.0%	44	39.6%	96	50.5%

[a] n = 62 Rotarix & breakthrough, n = 256 Rotarix & no breakthrough, n = 112 No Rotarix & RV diarrhea, n = 214 No Rotarix & no RV diarrhea

[b] n = 61 Rotarix & breakthrough, n = 247 Rotarix & no breakthrough, n = 111 No Rotarix & RV diarrhea, n = 190 No Rotarix & no RV diarrhea

Table S2. Association of vaccination with quantify of fecal viral shedding by qPCR among cases of RVGE defined by any rotavirus detection by qPCR

Analysis	n	Mean difference (95% CI), (log copies per gram of stool)	p-value for heterogeneity
Effect of Vaccination[a]	377	-0.79 (-1.17, -0.41)	-
Effect Modification by Severity[a]			
Mild	90	-0.79 (-1.64, 0.06)	REF
Moderate	117	-0.84 (-1.50, -0.18)	0.93
Severe	88	-0.75 (-1.63, 0.13)	0.95
Effect Modification by Age[b]			
< 6 months	143	-0.95 (-1.56, -0.34)	-
≥ 6 months	234	-0.68 (-1.19, -0.17)	0.51
Effect Modification by Doses Received[a]			
10wk – 19wk	66	-0.71 (-1.67, 0.26)	-
19wk – 1 yr	311	-0.81 (-1.25, -0.38)	0.85

[a] Controlling for age, WAZ, HAZ, exclusive breastfeeding at time of episode, and time (days) since symptom onset

[b] Controlling for WAZ, HAZ, exclusive breastfeeding at time of episode, and time (days) since symptom onset

Table S3. Association of vaccination with duration of illness in days among cases of RVGE defined by any rotavirus detection by qPCR

Analysis	n	Mean difference (95% CI), (days)	p-value for heterogeneity
Effect of Vaccination[a]	377	-0.09 (-0.71, 0.54)	-
Effect Modification by Severity[a]			
Mild	90	-0.37 (-0.87, 0.12)	REF
Moderate	117	0.04 (-0.83, 0.90)	0.41
Severe	88	1.36 (-0.56, 3.27)	0.09
Effect Modification by Age[b]			
< 6 months	143	0.08 (-1.23, 1.39)	-
≥ 6 months	234	-0.21 (-0.83, 0.41)	0.69
Effect Modification by Doses Received[a]			
10wk – 19wk	66	0.43 (-2.00, 2.86)	-
19wk – 1 yr	311	-0.27 (-0.84, 0.29)	0.59

[a] Controlling for age, WAZ, HAZ, and exclusive breastfeeding at time of episode

[b] Controlling for WAZ, HAZ, and exclusive breastfeeding at time of episode

Figure S1. Scatterplot of Age (days) at Time of RVGE Episode vs. Vaccine Status

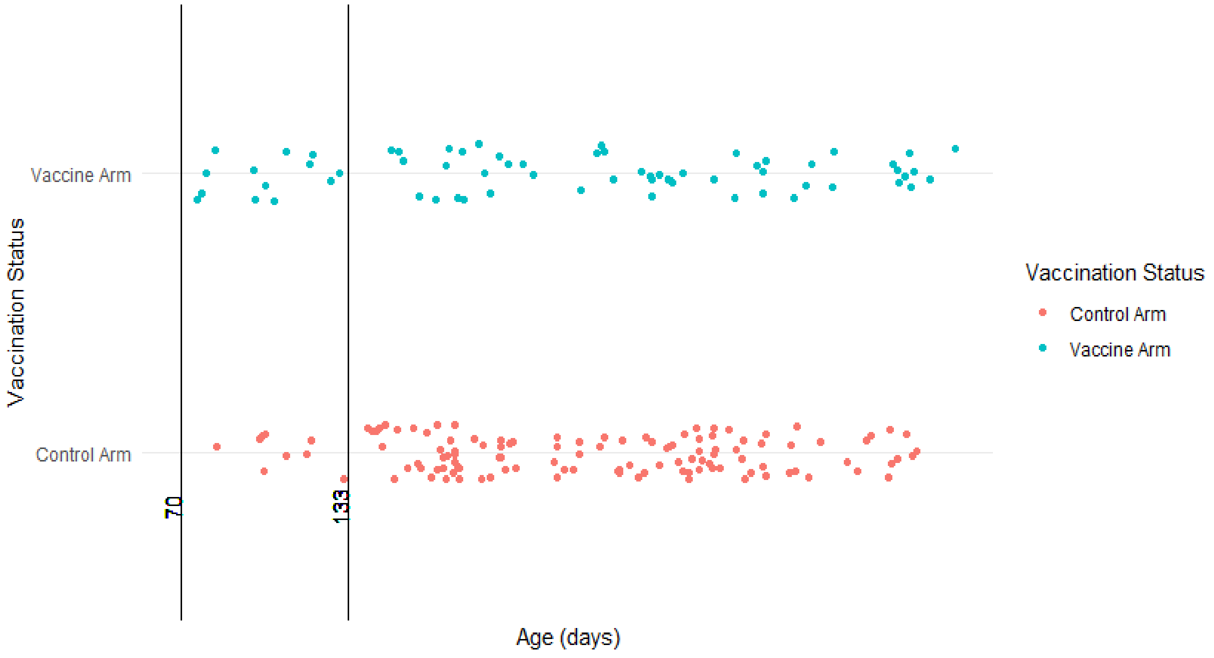

Supplement: jiad502_Supplementary_Data [file jiad502_supplementary_data.pdf]
